# Asháninka Palm Management and Domestication in the Peruvian Amazon

**DOI:** 10.1007/s10745-015-9745-1

**Published:** 2015-04-10

**Authors:** Joanna Sosnowska, Adam Walanus, Henrik Balslev

**Affiliations:** W. Szafer Institute of Botany, Polish Academy of Sciences, ul. Lubicz 46, 31-512 Cracow, Poland; Faculty of Geology, Geophysics and Environmental Protection, AGH University of Science and Technology, al. A. Mickiewicza 30, 30-059 Cracow, Poland; Research Group for Ecoinformatics and Biodiversity, Department of Bioscience, Aarhus University, Building 1540, Ny Munkegade 114, 8000 Aarhus C, Denmark

**Keywords:** Ethnobotany, Landscape domestication, Peru, Traditional ecological knowledge, *Arecaceae* palm cultivation

## Abstract

Palms are a natural resource that has been abundantly used by Amerindians for centuries. Only a few palm domestications have been reported in the American tropics, where there is great diversity of the *Arecaceae* family. We report the results of a survey combining ethnobotanical and ecological methods to study the past and present management and distribution of palms by the Asháninka indigenous people from the Tambo river region in the Peruvian Amazon. Our objectives were to document palm-related traditional ecological knowledge, to examine correlation between palm abundance and Asháninka management practices and social exchange of palm resources, and to address the question of how the Asháninka have modified palm diversity and distribution in their territory. We found that most palm species have multiple uses; the most intensively managed were palms that provide thatch, notably *Attalea phalerata*, *Oenocarpus mapora* and *Phytelephas macrocarpa*. Of these, *Attalea phalerata* was the most commonly cultivated and was found only in cultivated stands. Our results have implications for understanding the domestication of *Attalea weberbaueri,* which is a landrace within the *Attalea phalerata* complex. A closer understanding of this process would require morphometric and genetic methods to compare wild and managed populations.

## Introduction

Palms are common and diverse components of the Amazon rainforest, where palm communities may have up to 30–40 species per hectare with high density of individuals (Vormisto *et al.*^2004^; Montúfar and Pintaud 2006; Balslev *et al.*^2011^). At the same time, the great importance of palms for indigenous and Mestizo people in the Amazon basin has been demonstrated in numerous studies. Palms are possibly the plant family most abundantly used by the rural populations, because a number of different species provide thatch, housing materials, edible fruits and palm-hearts, oils, fibers, materials for the manufacture of domestic artifacts, tools for hunting and fishing, medicines and other products (e.g., Balick 1979, 1984; Plotkin and Balick 1984; Balslev and Barfod 1987; Borchsenius et al. 1998; Macía 2004; Sosnowska and Balslev 2009; Luziatelli *et al.*^2010^; Sosnowska *et al.*^2010^; Bussmann and Paniagua-Zambrana 2012). Palms are of key importance for subsistence strategies and cultural identity among many indigenous people (e.g., Schultes 1974; Balée 1988; Gertsch *et al.*^2002^; Byg and Balslev 2004). There have been many efforts to understand forest resilience and the effect of harvesting palm products from tropical forests (Balslev 2011; Bernal *et al.*^2011^; Brokamp *et al.*^2011^). Palm populations are managed in both sustainable and destructive ways, although while scientists often underline destructive human impacts on palm communities (Ruiz-Murrieta 1991; Hiraoka 1999; Castaño *et al*. 2007; Manzi and Coomes 2009; Bernal *et al.*^2011^), sustainable practices are rarely reported on.

Historical ecological research suggests that more than 12 % of the presumably pristine forests in the Amazon basin are anthropogenic in origin, and that without human intervention they would not exist in their present form (Balée 1989, 1994; Denevan 2008; Heckenberger 2010). According to Piperno (2011) in pre-ceramic occupation sites remains of only few palm genera have been documented, and these were possibly cultivated, although this cannot be empirically demonstrated with the botanical data.

Colonization of the Amazon basin by Europeans was more gradual than in the eastern coastal areas or the Andes, where indigenous populations were decimated through violence and disease in the sixteenth century. However, the Amazon’s dense tropical forests did not protect the indigenous inhabitants from the catastrophic effects of European colonialism, particularly diseases. At the time of first European contact Amazonia may have been populated by 4–5 million people who cultivated or managed at least 138 different plant species (Clement 1999), many of which were in advanced stages of domestication and human intervention was necessary for their maintenance. This crop genetic heritage was lost after 1492 through a 90–95 % decline of Amazonian Amerindian populations (Denevan 1992), which also resulted in expanding “fallow” forests that with time resembled untouched tropical forest (Heckenberger 2010).

Domestication is a process that produces plant populations better adapted to cultivation, but at the same time they lose ecological adaptation to their wild habitat. For example, seed crops under cultivation lose their dispersal capacity. Also, moving horticultural plants to new environments, which makes them dependent on human intervention, is termed domestication (Ladizinsky 1998).

Detailed studies of landscape changes caused by the Kayapó and Ka’apor in Brazil (Posey 1985; Posey and Balée 1989; Balée 1994), the Huaorani in Ecuador (Rival 1996) and the Nukak in Colombia (Politis 1996) have shown how anthropogenic palm groves can modify forest landscapes. These studies demonstrate that human influence can enhance rather than reduce palm diversity. However, although traditional farmers also manipulate the forest surrounding their settlements, all these studies concern only Amazonian hunter-gatherers. The cumulative impact of their small bands, on the basis of available evidence (Stahl 2008), is greater than that of agriculturalists.

Environmental manipulation by human societies, who consciously or unconsciously manage plant populations and animal groups, leaves traces in the landscape even long after they occurred (Harlan 1992; Harris 2012). According to Clement (2013):

Landscape domestication is a process in which human intervention in the landscape and manipulation of landscape components results in changes in landscape ecology and in the demographics of its plant and animal populations, resulting in a landscape more productive and congenial for humans.

How does human influence on palm diversity look in the case of indigenous Amazonian agriculturalists? We found Asháninka palm management to be an unexplored but meaningful example of landscape domestication.

Posey (1992) argued for a move away from the “archaic dichotomy” between what is “natural” and what is “cultural” and that both natural and social scientists should adopt a historical perspective in studying people and landscapes in the Amazon. He proposed using a distinction between *etic* and *emic* explanations of cultural phenomena: “Emic interpretations reflect the cognitive and linguistic categories of the natives, whereas etic interpretations are those that have been developed by the researcher for purposes of analysis” (1992).

We use the emic-etic distinction to investigate the cultural domain of palms among the Asháninka through a combination of ethnobotanical and ecological methods. Specifically we investigated palm-human interactions from two perspectives: the importance of palm resources to the human society, and the impact of humans on palm communities. Our objectives were to document palm-related traditional ecological knowledge (TEK), to examine any correlation between palm abundance and Asháninka management practices and social exchange of palm resources, and to address the question of how Asháninka activities have modified palm diversity and distribution in their territory. We assumed that the most frequently cited palms represent the most intensively managed species in both surrounding forest and in home gardens. We expected there would be decreasing abundance and diversity of natural palm resources in the surrounding forest and increasing abundance and palm diversity in home gardens.

## Methods

### The Asháninka and the Study Area

**Fig. 1:**
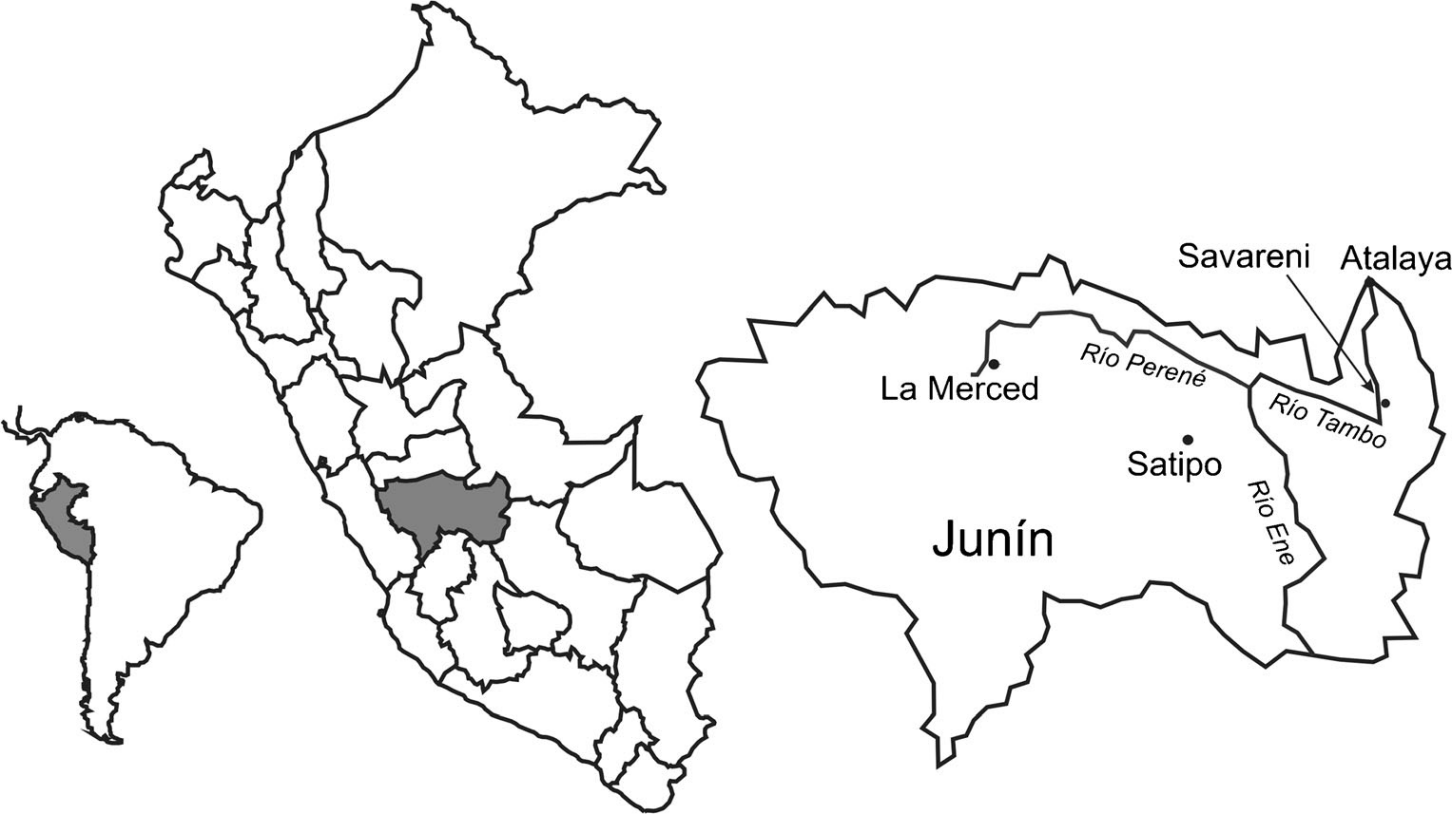
Location of Savareni village in the study area in the department of Junín in Peru

The Asháninka make up 26 % of the indigenous population in the Peruvian Amazon and are the largest group of the Arawak language family, which in Peru also includes Amuesha (Yánesha), Ashéninka, Caquinte, Culina, Piro (Yine), Nomatsigenka and Matsiguenka (Santos-Granero and Barclay 2005). Of the total population of 90,000 Asháninka, 65,000 live in the department of Junín (INEI 2009). Our study site is the village Savareni in the Tambo District, Satipo Province, in the Department of Junín (11°13′S; 73°41′W; Fig. 1).

The closest town is Atalaya, 57 km away as the crow flies and a little more along the Tambo River. The village of Savareni lies at 270 m a.s.l. and is an annex of the larger community of Poyeni, which covers an area 10,953 ha (Benavides 2006). The climate corresponds to that of tropical-humid forest in the Holdridge (1976) classification. Mean annual precipitation is 1500 mm, with a wet season from September–April and a dry season from May–August. The average monthly temperature varies from 25 to 27 °C, with a minimum of 18 °C and a maximum of 36 °C, and humidity oscillates around 75 % (Rodríguez 2008).

The 170 inhabitants of Savareni live in 20 family groups near a lagoon of the Tambo River. Because of the rocky soil around the village most fields are situated on the more fertile island on the other side of the lagoon. The remaining six settlements are located deeper in the forest at a lower density, mainly near the Shicapaja stream. Every household has at least two separate buildings: a dormitory and a kitchen. Most maintain secondary homes near their fields at some distance from the main community. On the other hand some families living near the Shicapaja stream have built their secondary homes in the village so that their children can study at the Savareni primary school.

Asháninka swidden agriculture is characterized by a relatively small area of forest disturbance, multi-cropping, great genetic diversity of crop cultivars, and rapid forest regeneration (Posey and Balée 1989). The Asháninka from the Tambo region use the slash-and-burn method to clear lands, mostly for subsistence agriculture. The most important crops are yuca (*Manihot esculenta* Crantz), corn (*Zea mays* L.), bananas (*Musa paradisiaca* L.), beans (*Phaseolus* sp.) and rice (*Oryza* sp.). Small-scale cacao (*Theobroma cacao* L.) plantations generate additional income. Hunting and fishing are also important to Asháninka livelihoods.

### Ethnobotanical Field Data

An initial visit to Savareni village took place in December 2008. Data sampling was carried out from October 2009–February 2010 and from May–July 2011. Data was collected mainly with use of the free listing technique (Alexiades 1996; Martin 2007).

During October 2009–February 2010, a free-listing survey was administered to 50 informants, who were asked to name in Asháninka or Spanish (the language was up to them) all the palms they recognized. Based on the list compiled from their responses, a second question was posed regarding uses for each palm from the list. Because some use categories established a priori were unclear to the respondents, we decided to adopt the Asháninka classification of palm uses. Additionally, direct questions were asked about palm characteristics, extraction methods, habitat, forest types, etc.

During May–July 2011, we used free listing to identify the cultivated palms. Information was also collected during informal conversation and by asking direct questions referring mainly to the motivation of the respondents for cultivating palms on their land, their perception of variation, their preference of palm traits and their management practices.

Interviews concerning different topics were often conducted with the same informants; in total 71 people were interviewed. The age range of the informants was 18–89 years and the gender distribution was 46 males and 25 females.

### Data Analysis

For the statistical analysis, free-listing uses were tabulated with scientific palm names and use categories established a priori: food (fruits, palm hearts, oil, chicha drink, *emoki* larvae); construction material (thatch, house posts, floor and walls); tools (e.g., bows and spears, mats and fans, baskets and weaving tools); medicinal functions and ornaments. Data were analysed according to the methods proposed by Ryan *et al.* (2000). Folk palm names that occurred more often were assumed to be more salient in the cultural domain. An additional measure of salience was calculated by considering the order of mention of folk palm names. Congregated data from 50 interviews was analysed by the use of correspondence analysis (CA) in Statistica v. 10. Correspondence analysis is a practical technique for exploring and describing tables of categorical data (Greenacre 1984; Weller and Romney 1990; Ryan *et al.*^2000^; for an exceptionally clear description of the technique see Watts 1997). The technique scales the rows and columns of a table into the same multidimensional space. It can be used on both large and small samples and is relatively insensitive to cells with low or no values.

### Ecological Data

To determine the abundance of individual species of palms in the forest surrounding Savareni village we made four transects. Each transect was 500 m long and five meters wide. This size of transect includes almost all palm species in a uniform segment of forest (Balslev *et al.*^2010^). In subunits of 5 × 5 m we identified and counted all palm individuals.

We placed the transects at different distances from the village correlated with degree of human impact. We expected that accessibility of palm resources would decrease further away from the village, and that palm abundance and diversity would be higher in the more distant forest transects. The first transect was placed in the forest about one km from the current Savareni village.^1^ The second transect was located on the western side of Río Tambo in the forest close to the former Savareni village. The third transect was in the forest on rocky soil close to the eastern riverbank of Río Tambo. Transect number four was in the forest 10 km from the current Savareni village.

Palm distribution was estimated by comparison of four forest transects and a fifth transect established *post hoc* based on an earlier prepared map in which all the palm individuals in Savareni village had been drawn to their exact location in home gardens and fields.

Palms were identified in the field using the *Field guide to the palms of the Americas* (Henderson *et al*. 1995). Specimens were packed in plastic bags, moistened with 70 % alcohol and subsequently pressed and dried in the herbarium of the Museum of Natural History UNSM, Lima. In total 72 voucher specimens were deposited in herbarium KRAM in Poland with duplicates in herbarium MHN in Peru; herbarium acronyms as in (Thiers 2010). The nomenclature of palm names and the authors’ names were updated to follow the *World Checklist of Palms* (Govaerts and Dransfield 2005).

## Results

### The Importance of Palms for the Asháninka

**Fig. 2:**
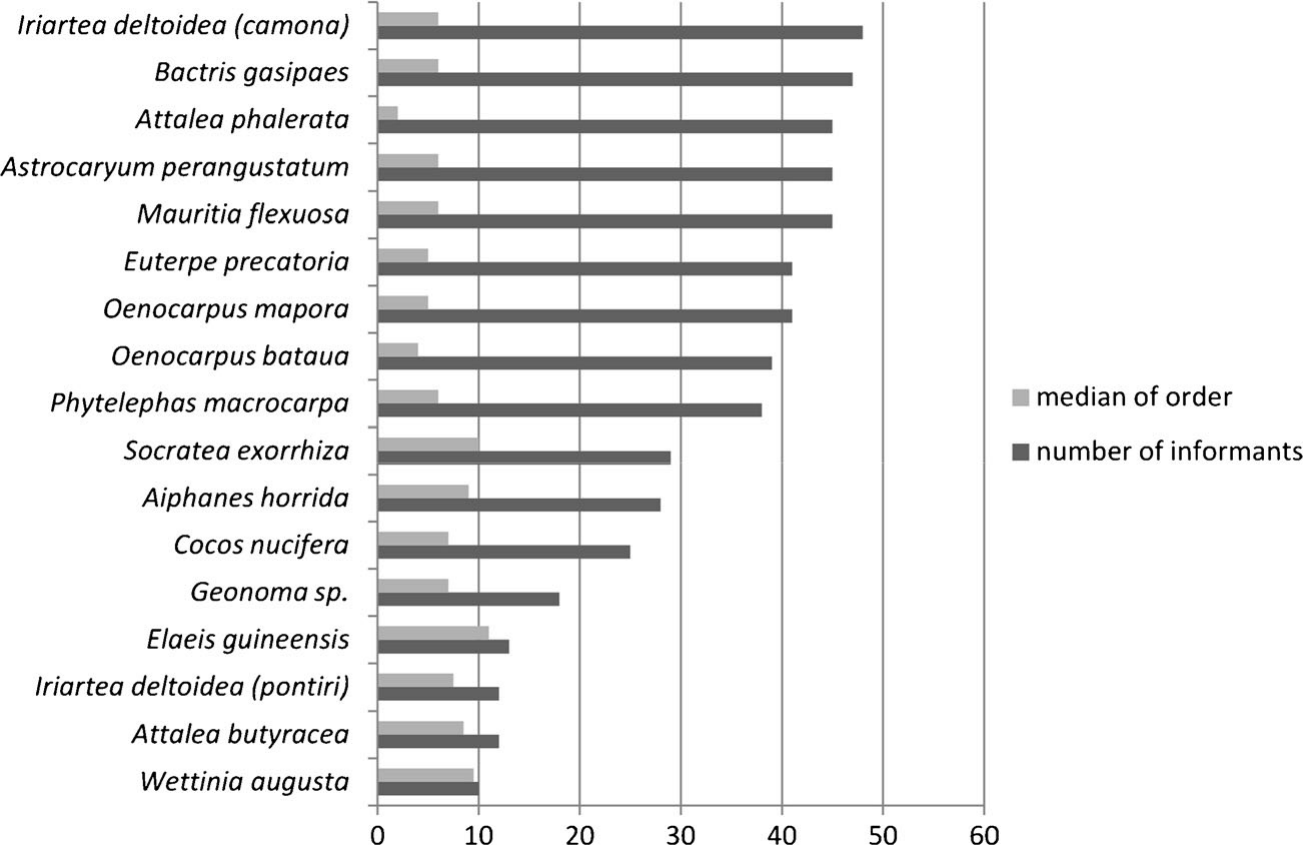
The most important palm species for Asháninka people from the Tambo region according to the relative saliency of their folk names

The free lists of folk palm names contained 6–15 palm species for each respondent. Most mentioned *camona*, but only a few mentioned *pontiri*—both recognized by Asháninka as two different types of *Iriartea deltoidea. Pontiri* is distinguished as a separate folk species, because of substantial morphological differences. The upper part of the *pontiri* stem is not swollen and the stem diameter is smaller than *camona*, from which only 4–5 palm individuals are needed for the floor of one house. For this reason *pontiri* is not recommended for floor construction. Another important species in the construction category—*Attalea phalerata* (*tsiaro*)—of which the leaves are preferred for thatch, was ranked third (Fig. 2). However, *tsiaro* had the lowest median of order, which means that many respondents listed the name as the most important first item in an (emic) Asháninka valuation (Table 1).

**Table 1:** Asháninka useful palms listed according to median of order and number of respondents who mentioned these palms and their emic use categories

Latin name	Asháninka folk name	Median of order	Number of respondents	Asháninka *emic* use categories
*Attalea phalerata*	*tsiaro*	2	45	Fruits, palm hearts, oil, *emoki* larvae; thatch; mats and fans, baskets and tools; ornaments
*Oenocarpus bataua*	*shaki*	4	39	Fruits, palm hearts, oil, *chicha* drink, *emoki* larvae; thatch, house posts; mats and fans, baskets; medicaments; ornaments
*Oenocarpus mapora*	*chorina*	5	41	Fruits, palm hearts, *chicha* drink, *emoki* larvae; thatch, house posts; mats and fans, baskets; medicaments
*Euterpe precatoria*	*tsirentsi*	5	41	Fruits, palm hearts, *chicha* drink; house posts; mats and fans; medicaments, ornaments
*Iriartea deltoidea*	*camona*	6	48	Thatch, house posts, floor and walls; tools, ornaments
*Bactris gasipaes*	*kiri*	6	47	Fruits, palm hearts, *chicha* drink, *emoki* larvae; walls; bows and spears, waving and another tools like hunter traps, raft and roofing nails; medicaments
*Astrocaryum perangustatum*	*tiroti*	6	45	Fruits, palm hearts, *emoki* larvae; thatch, house posts; mats and fans, baskets; ornaments
*Mauritia flexuosa*	*toniro*	6	45	Fruits, oil, *chicha* drink, *emoki* larvae; ornaments
*Phytelephas macrocarpa*	*compiro*	6	38	Fruits, palm hearts; thatch; medicaments; ornaments

A free list of uses showed Asháninka use categories, including the specific part of the palm used. The order in which informants presented their answers was considered when collating the results from the corresponding free lists of palm names and free lists of uses by palm parts.

**Fig. 3:**
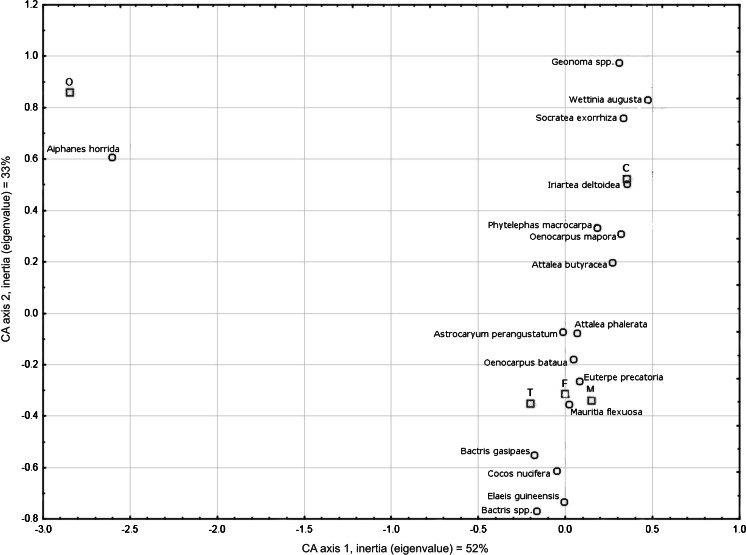
Correspondence analysis of palm species with use categories (*C* construction materials, *F* food, *T* tools, *M* medical preparations, *O* ornaments)

Palms are rarely used as ornaments, a use associated with only one species—*Aiphanes horrida,* which seems to be used almost exclusively for this purpose (Fig. 3). Some species of *Geonoma*, *Wettinia augusta*, *Socratea exorrhiza* and *Iriartea deltoidea* are strongly associated only with the construction category. The most versatile species appear in the central part of the graph, showing associations with different categories represented by relative distance between species and items of categories (Fig. 3). *Euterpe precatoria* and *Mauritia flexuosa* are strongly associated only with food and medicinal functions. *Bactris gasipaes* is distinct from the other most useful palms because it does not correlate with construction materials. It is worth mentioning that the relative distance of *Bactris gasipaes* to tools is closer than to food. That suggests that using *Bactris gasipaes* wood for making bows, arrowheads and tools to weave cotton is more important, or at least as important, as obtaining edible fruits from this palm.

**Fig. 4:**
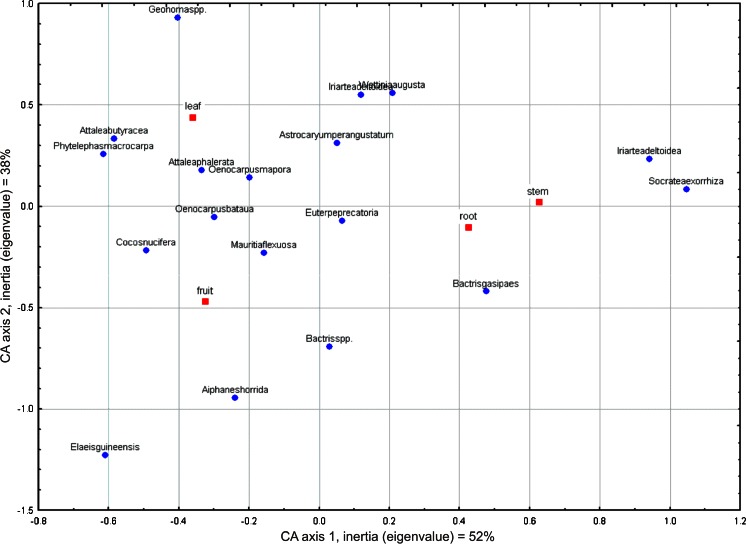
Correspondence analysis of palm names and uses by palm part, in order of mention by informants

The uses of *Iriartea deltoidea* and *Socratea exorrhiza* are based exclusively on their ‘stems.’ Especially useful *camona* wood obtained from *Iriartea deltoidea* is the most common material for floor construction. *Iriartea deltoidea* and *Socratea exorrhiza* are distinct from the other species, which tend to cluster together between the ‘fruit’ and ‘leaf’ categories. Also domesticated *Bactris gasipaes* strongly corresponds with a ‘stem’ and its wood is used mainly for making of various tools (Fig. 3) important for subsistence (Fig. 4). The *kiri* (*Bactris gasipaes*) in the Asháninka interpretation corresponds more with the ‘stem’ than the ‘fruit.’ However, the edible *kiri* fruits are also highly appreciated by the Asháninka.

Species of *Bactris*, *Aiphanes horrida* and *Elaeis guineensis* correspond to the ‘fruit’ but other palms whose fruits are eaten are notable. The importance of these species for the Asháninka is limited, and the fruits are the only useful part of these palms. The fruits of *Aiphanes horrida* are strongly associated with ‘ornaments’ (Fig. 3) because they are a very common source of seeds used to decorate female *cushma*—a traditional cloth. *Elaeis guineensis* is an alien species that has not been as greatly incorporated into either social or cultural usage as *coco*—*Cocos nucifera*—another introduced species.

*Geonoma* species are understory palms with simple or pinnate leaves, with only a few leaflets, which are used by Asháninka from the Tambo region as thatching material. However, in Savareni the houses are mainly thatched with long pinnate leaves with numerous leaflets from such as species of *Attalea, Oenocarpus* and *Phytelephas macrocarpa*. Most palms were multiple-use, versatile species, whose different parts are used in more than one use category.

### Distribution and Social Exchange of Palm Resources in the Tambo Region

**Fig. 5:**
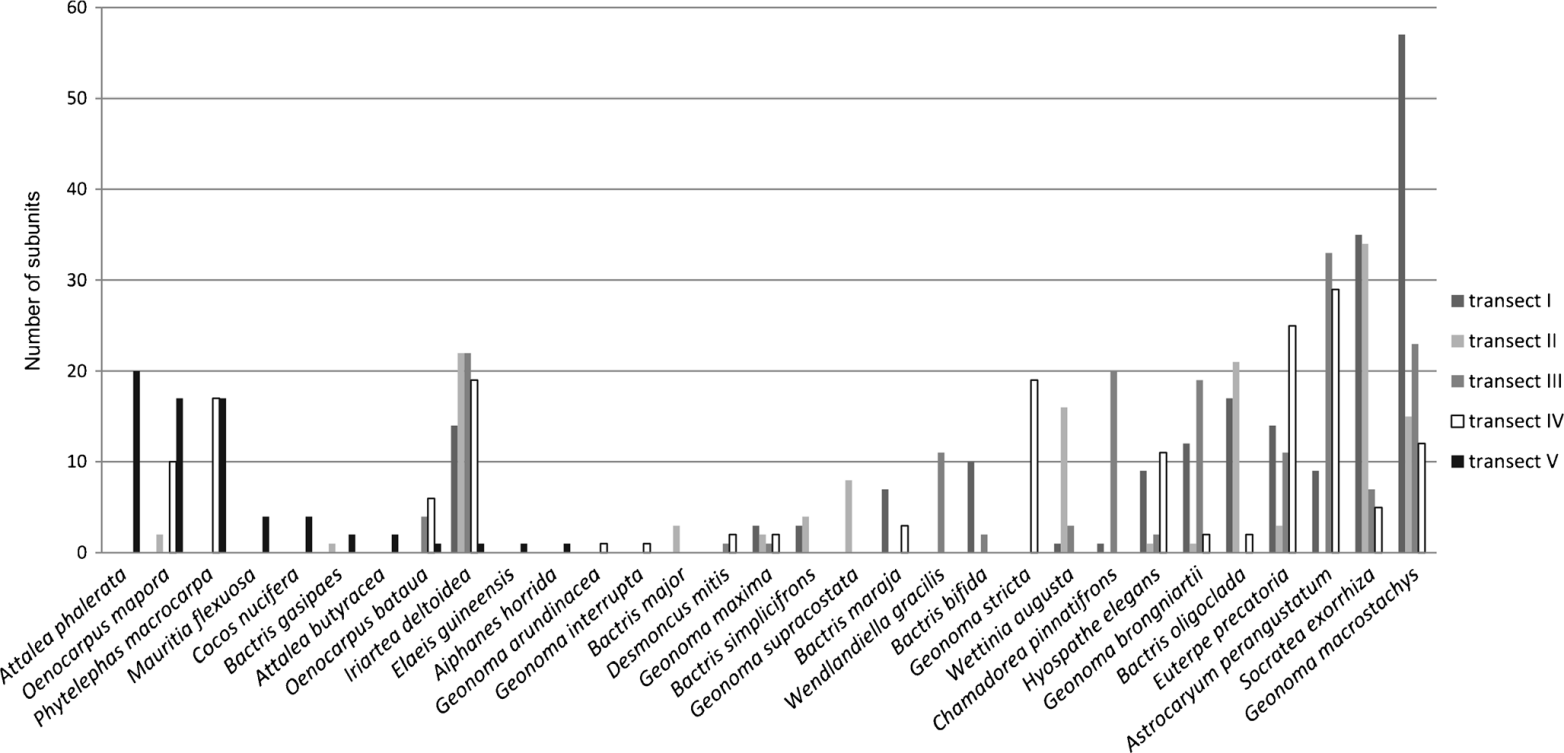
Comparison of palm distribution among transects by number of transect subunits with presented palm species individuals (without seedlings)

**Fig. 6:**
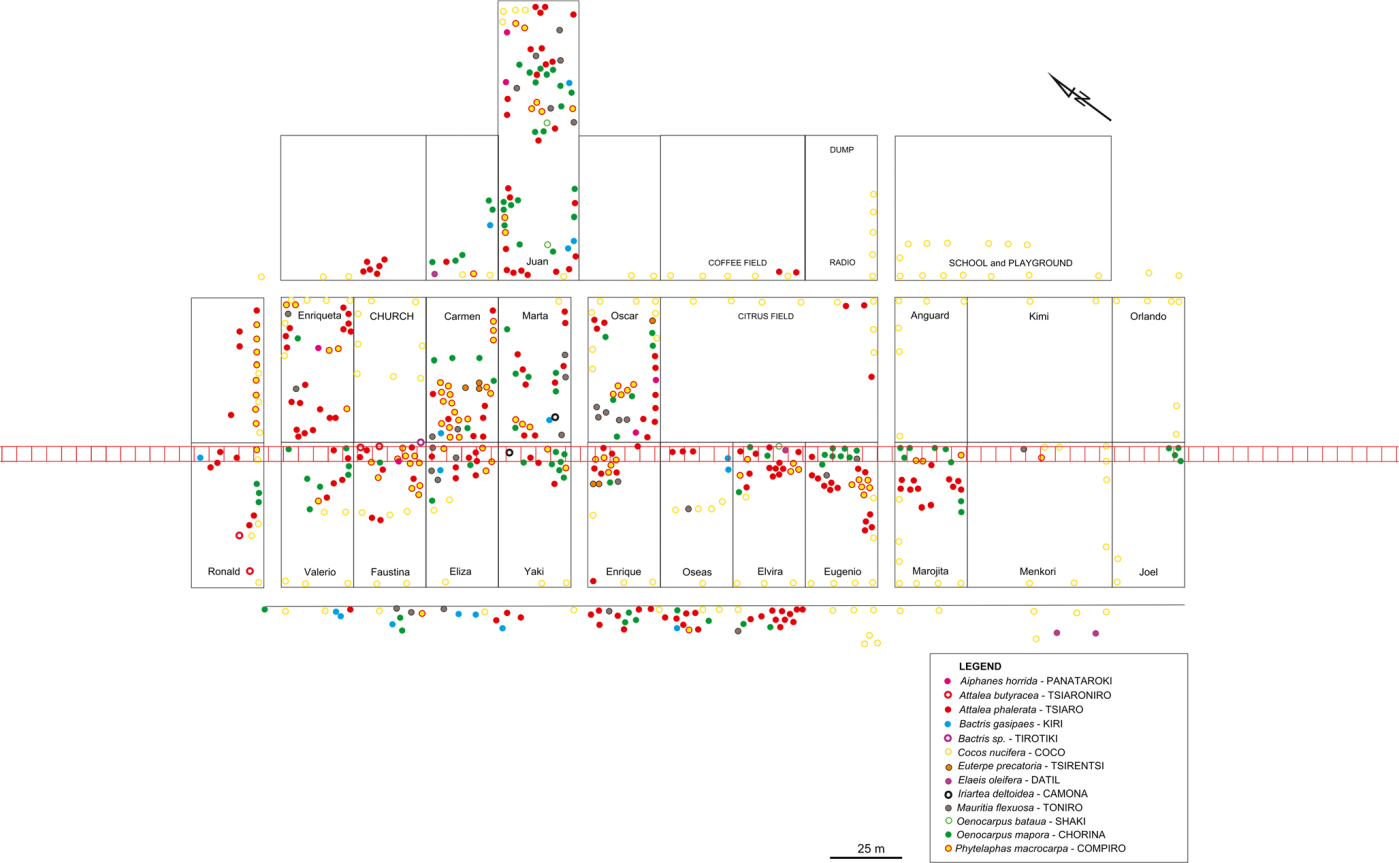
Palms planted in Savareni home gardens

We identified 34 species of palm during fieldwork in the Tambo region. Thirty were recorded in the transects we established in different environments around Savareni (Fig. 5). In transect V within Savareni we recorded 11 palm species, of which six were found only in the village transect (Fig. 6).

*Attalea phalerata* was the most abundant palm species in Savareni (Table 2). All individuals of *Attalea phalerata* growing in the village derive from the same common ancestral plant according to the eldest female informant (the founder of Savareni, and conveyed during informal conversation). After a visit to her daughter, who lived in the Asháninka community located along the Ucayali River close to the city of Atalaya, she brought back four *Attalea phalerata* seeds, whose leaves (*tsiaroshi)* her daughter had recommended as excellent thatching material. Three of them germinated in her home garden and survived to their fruiting time. The her daughter-in-law commented during the conversation:

**Table 2:** Cultivated palms in Asháninka home-gardens in Savareni village

Latin name	Asháninka folk name	No. of individuals in Savareni	Availability of useful palms and its distribution in the forest	Primary events of planting
*Attalea phalerata*	*tsiaro*	189	Cultivated only in home gardens and fields, absent in the surrounding forest	Seeds brought around 30 years ago from relatives lived in Ucayali river region
*Cocos nucifera*	*coco*	161	Cultivated only in homegardens, introduced species absent in the forest	Planted before 1993 by previous inhabitants of the site
*Oenocarpus mapora*	*chorina*	97	Cultivated in home gardens, limited availability in the forest, present mainly in further distance from the village	Seeds planted from the forest
*Phytelephas macrocarpa*	*compiro*	87	Cultivated in the fields and home gardens, found only in a remote forest	Seeds brought in 1990’ from relatives of Oviri and Anapate villages in Tambo region
*Mauritia flexuosa*	*toniro*	32	Cultivated in home gardens and swamp forest, absent in the surrounding forest	Seeds brought from Betania, Mayapo and Poyeni villages in Tambo region
*Bactris gasipaes*	*kiri*	17	Cultivated in the fields and home gardens, found also in the secondary forest in previous location of Savareni village, but absent in the forest	Seeds brought from previous location of Savareni village
*Euterpe precatoria*	*tsirentsi*	6	Common in the surrounding forest, rarely cultivated in home gardens	Seeds brought from the forest
*Aiphanes horrida*	*panataroki*	6	Absent in the surrounding forest, rarely cultivated in home gardens	Seeds brought from the remote forest
*Attalea butyracea*	*tsiaroniro*	4	Absent in the surrounding forest, rarely cultivated in the fields and home gardens	Seeds brought from Capitiri and Shevoja villages in Tambo region
*Oenocarpus bataua*	*sha*	3	Cultivated in the fields and home gardens, found only in a remote forest	Above 5 years ago seeds rarely seedlings from remote forest started to be planted

My mother-in-law used to invite us for eating *tsiaroki* when the fruits are ripe. My son said: Let’s plant *tsiaro* for our own stock of seedlings (…) to continue planting more to produce more and more *tsiaro*.

**Fig. 7:**
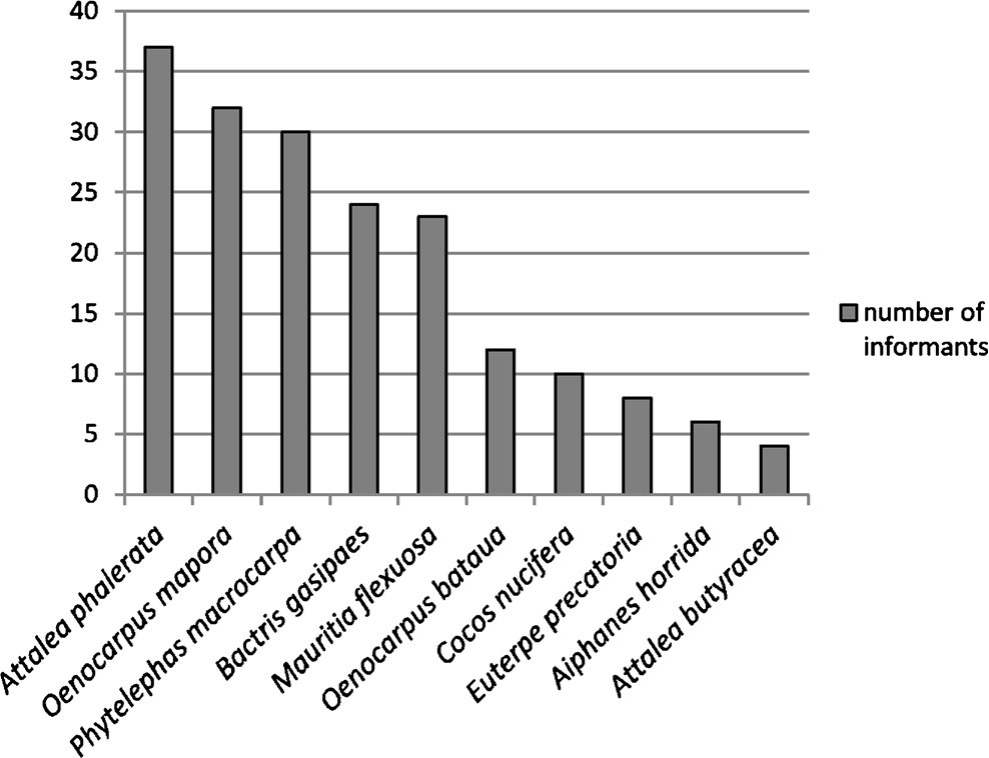
Palm species mentioned as planted by respondents themselves (45 informants)

**Fig. 8:**
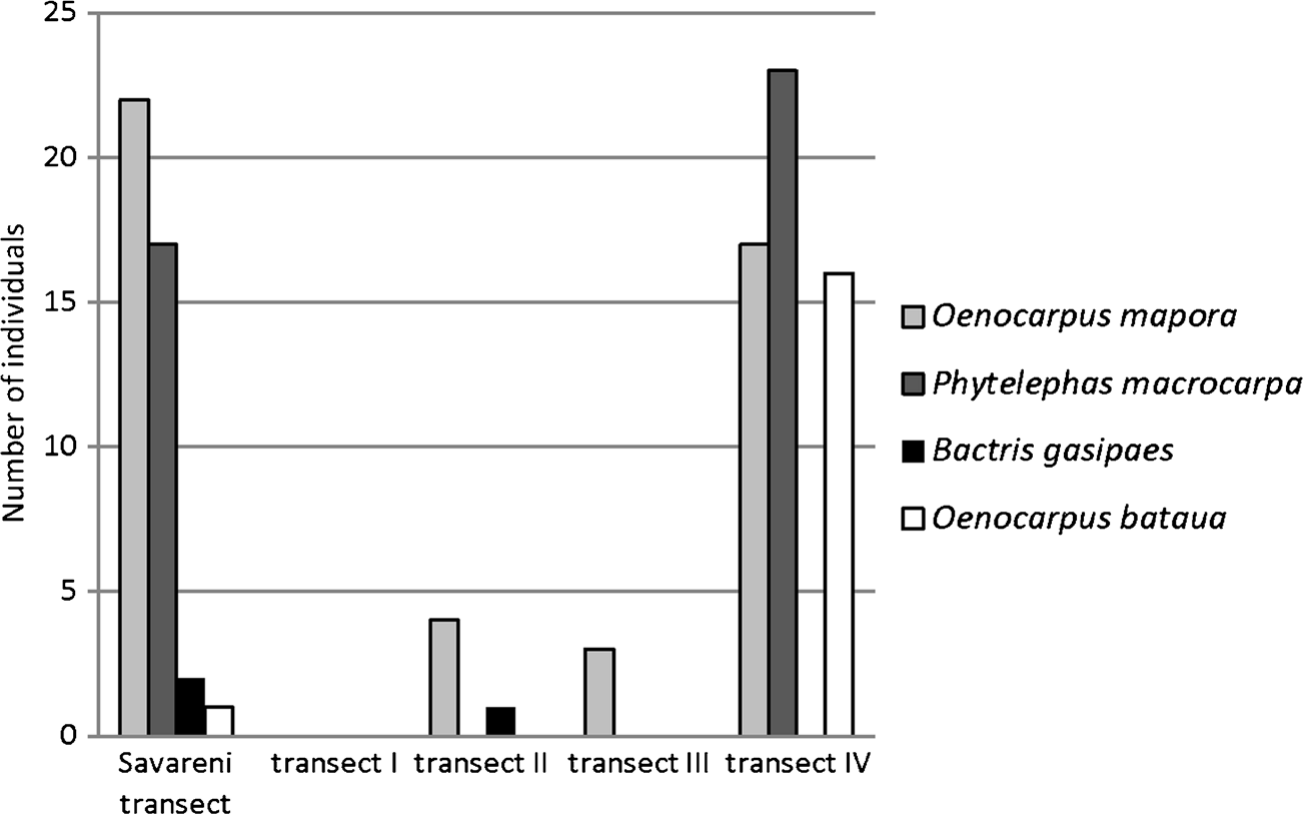
Comparison of common palm species from the village and forest transects by number of individuals in each transect

The question “Have you ever planted a palm?” elicited examples of half-conscious planting through eating fruits and throwing the seeds on the ground. Of the 45 informants who were asked the question, all answered positively, with a median of four different palm species (Fig. 7). *Bactris gasipaes, Oenocarpus bataua, Oenocarpus mapora* and *Phytelephas macrocarpa* were present both in the village and in at least one of the forest transects (Fig. 8).

The stock of seedlings located farthest away in the study area was 3–4 h walking distance from the closest village. Our Asháninka guide drew attention to *Oenocarpus bataua*, which was growing on the hardly visible path. The palm was very tall and it was not possible to see its reproductive structures hidden high up in the canopy. There were no seeds or seedlings on the ground, but the guide claimed that the palm already had fruited many times:

This *shaki* grows on the path. People from Poyeni walking this way pick up all seeds and seedlings to plant them near their houses.

*Phytelephas macrocarpa* was a novelty in Savareni village in the 1990s. A son of our elderly female informant brought the first fruits from Oviri village and shortly afterwards he and his wife brought a basket of *compiroki*—seeds of *Phytelephas macrocarpa*—from Anapate village, a gift from one of the wife’s uncles, and many were planted in Savareni. However, the seeds were always obtained from the *Phytelephas macrocarpa* that grew in the forest without human intervention in both Anapate and Oviri villages.

### Asháninka Palm Management and Landscape Domestication

The main reason for planting palms in home gardens is for their leaves, which are used for thatch. As one informant noted:

Rather than carrying leaves for 2 h from the forest it is better to pick up seeds for planting in the field. When it grows we acquire leaves for thatching.

**Fig. 9:**
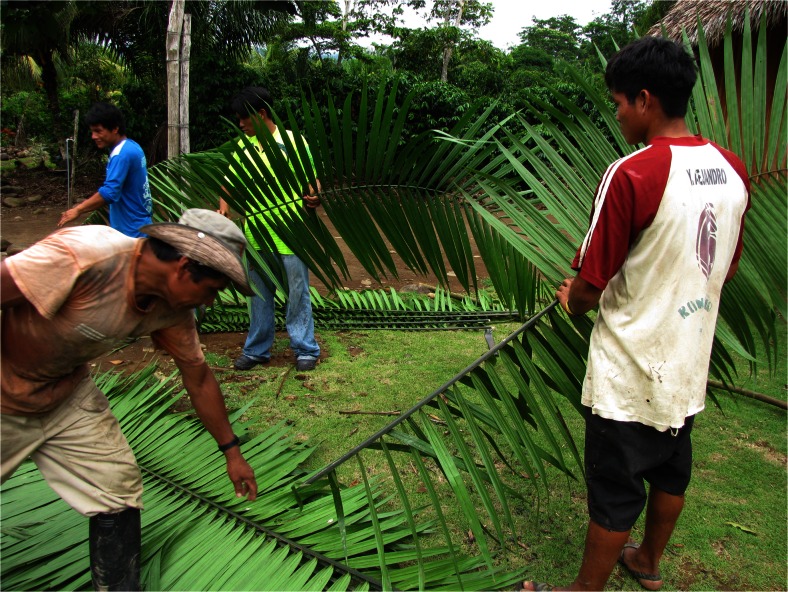
Preparation of *chorinashi* - *Oenocarpus mapora* leaves for repair of the radio station roof

For roof repairs, every family has enough leaves from their own palms, but new house construction requires cooperation with neighbours or planning over a long period. Communal buildings with palm roofs are repaired by all inhabitants of the village during the regular Friday *faena*—communal work session (Fig. 9).

Depending on the level of familiarity and trust, neighbours lend or sell leaves from their palms. During the harvest 4–5 leaves are left on each individual, which enables the palm to continue to produce more leaves. Villagers plant palms behind their houses in the village as well as in their fields. Usually palms are planted only along the edge of fields because they are believed to draw too much water, which is damaging for other more sensitive crops. When the couple that brought the first basket of *Phytelephas macrocarpa* seeds from Anapate village planted them in their field of coffee plants they did not produce a satisfactory crop.^2^

To thatch a 12 × 4 m guest-house, around 3000 *compiroshi - Phytelephas macrocarpa -* leaves are required, but for the same size house only about 1000 *tsiaroshi* - *Attalea phalerata –* leaves are needed (see Tables 2 and 3).

**Table 3:** Comparison of two palm species brought to Savareni village for thatching material recommended by family members living in other villages

*Attalea phalerata - Tsiaro*	*Phytelephas macrocarpa - Compiro*
Seeds planted with a bigger space gap	Seeds planted, 3 in one seedbed
Germinates after a year and grows slowly	Germinates after a year and grows slowly
The palm takes up more space and makes more shade for other plants	Doesn’t grow so big and doesn’t make too much shade for other plants
Leaves are longer, less of them are needed to cover a roof	Leaves are shorter, three times more of them are needed to cover a roof
Heavy leaves, difficult to lift to the roof	Smaller, lightweight leaves easier to lift
The roof is finished faster but with more hard work	Making a roof takes more time but is not such tedious work
Leaflets are broader and more resistant, they start to crack after a few years	Leaflets are narrow and less resistant, they start to crack after half a year
Roof thatch lasts for 9–10 years	Roof thatch lasts for 7–8 years

**Fig. 10:**
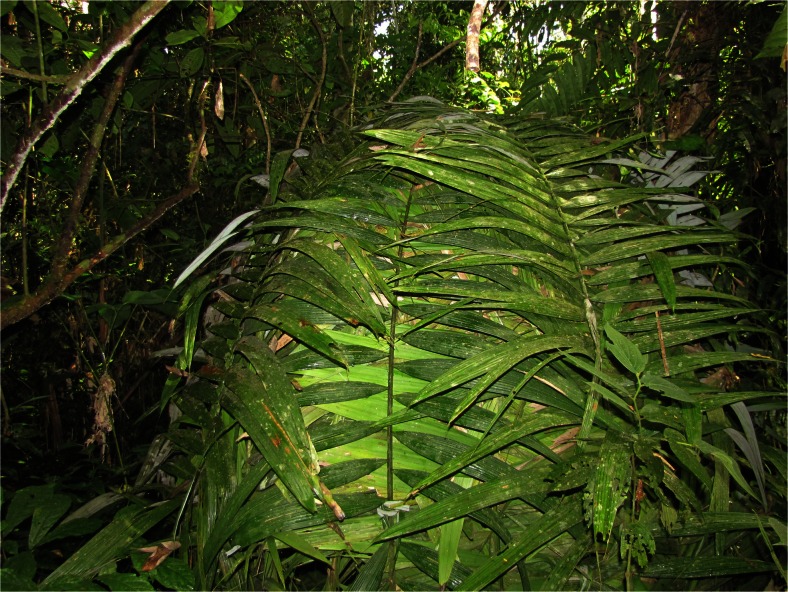
Traditional hunter shelter from *chorinashi* - *Oenocarpus mapora* leaves

**Fig. 11:**
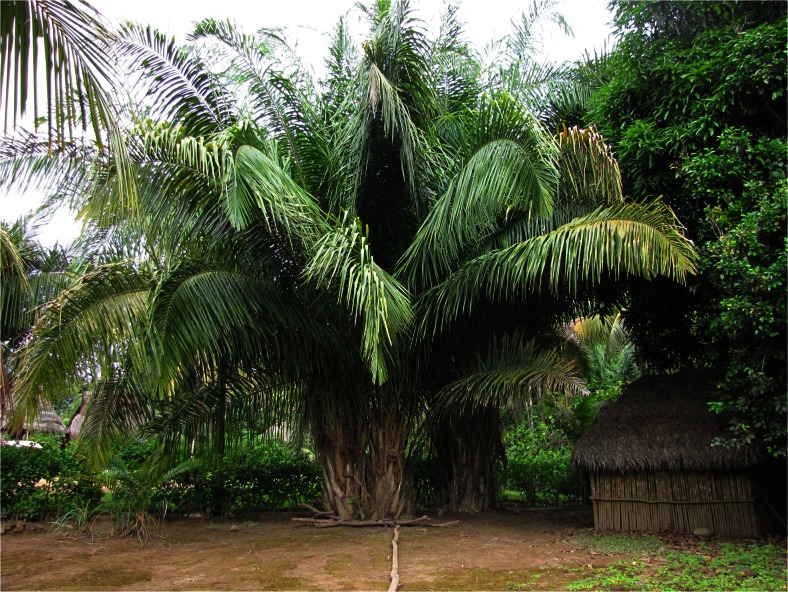
*Attalea phalerata* – *tsiaro* individual in Savareni village

*Oenocarpus mapora*, *chorina*, which as well as leaves for thatch, has a tall stem used for construction as house posts, is usually harvested by cutting down the entire palm. However, in general not all stems of the individual clumps are cut down and new suckers called *obeshiki* grow fast. The leaves of *chorina* are considered less resilient than those of the other species mentioned by respondents, lasting only 6–7 years. Palm leaves are also used to make various temporary roofs, such as panels of *Attalea phalerata* to shade cacao saplings on a new plantation or a traditional hunter shelter in the forest made from *Oenocarpus mapora* leaves (Fig. 10). The ancestral individuals of the abundant *Attalea phalerata* population in Savareni village themselves came from cultivated fields near Atalaya. Individuals growing in Savareni village had wide leaflets and developed only short aerial stems (Fig. 11).

**Fig. 12:**
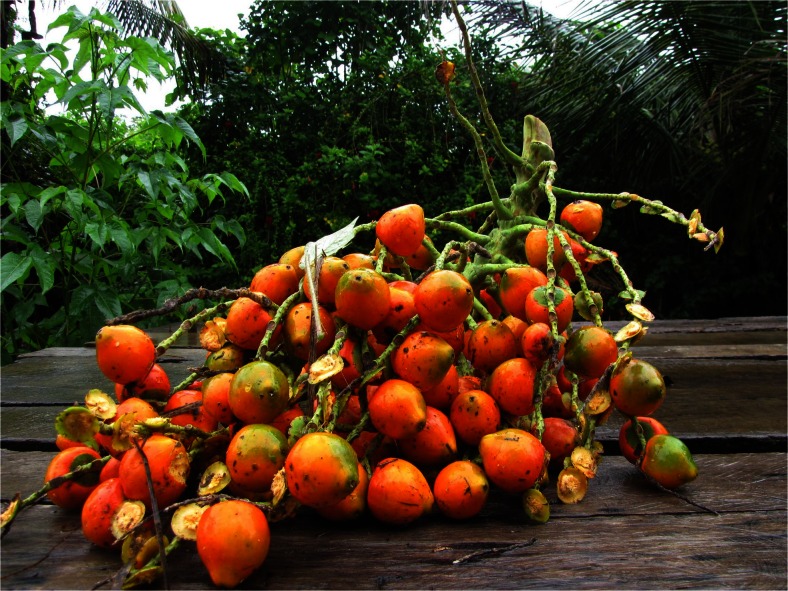
*Kiriki* - fruits of *Bactris gasipaes*

The second reason for planting palms that was mentioned by the Asháninka is their edible fruits (Fig. 12). Palms planted mainly for their fruits were: *toniro* (*Mauritia flexuosa*), *kiri* (*Bactris gasipaes*) and *tsirentsi* (*Euterpe precatoria*). In our study area, where *Mauritia flexuosa* did not grow without human intervention, every planted *toniro* is individually owned and the fruit is harvested with the use of a ladder or a pole. However both male and female individuals that do not produce fruits well are cut down for breeding *emoki* (*Rhynchophorus palmarum*) larvae. On a trip to collect *tsirentsi* (*Euterpe precatoria*)*,* which grows in the swamp near the village, our Asháninka guide explained that he planned to plant *toniro* in the swamp rather than his home garden because it was a natural place where the palm grows better.

*Euterpe precatoria* fruit is usually harvested by climbing the palm. Spiny *Bactris gasipaes* fruits are also harvested in a manner ensuring the survival of the palm, by putting another palm stem against it or reaching them with a pole. When asked about practices associated with planting palms, Asháninka respondents usually mentioned the elimination of other plants growing near the seedlings. Clearing of undesirable plants and dry leaves is also practiced in adult cultivated palm stands during the harvest of leaves for roof thatch.

## Discussion

Many of the early agriculturalists in the Amazon basin were of the Arawak family (Hill and Santos-Granero 2002), of which the Asháninka form the largest group today. Many areas from the southern Amazon basin that were dominated by speakers of Arawak languages in the past show evidence of essential pre-Columbian modifications in the landscape such as earthworks and residential sites (Heckenberger *et al.*^2003^, ^2008^; Hornborg 2005; Heckenberger 2010).

The Amazon basin agriculturalists probably relied more heavily on fruit crops than those in other areas in the Americas (Patiño 1963). *Bactris gasipaes* may have been one of the basic crops in the region, based on its degree of domestication (Clement 1988) and its importance to many societies, as reflected in their legends, myths, ceremonies and festivals (Patiño 1992; Santos-Granero 2011; Sosnowska and Kujawska 2014). Patiño (1989), discussing the idea that *Bactris gasipaes* was domesticated because of its starchy fruits (Sauer 1952), suggested that the initial impetus might have been use of the wood, which nowadays is preferred for technological artifacts in many areas (Rival 1996). According to Bellwood (2005), species with technological uses may have been among the first to be domesticated because they were often essential for hunter-gatherers’ subsistence. Respondents in our study emphasized more strongly the utility of *Bactris gasipaes* palms to the ‘stem’ and suitability for making ‘tools’ than to ‘fruit’ and ‘food.’ This tendency is confirmed by correspondence analysis of *Bactris gasipaes* both to use category (Fig. 3) and to palm part (Fig. 4).

Posey (1992) noted the Kayapó in Brazil regarded only plants that could not grow without the help of humans as “planted”; all other species were considered as “natural.” However, the “planting” concept of Asháninka informants in the study area of the Tambo region in Peru was less restrictive.

Genetic selection among thousands of species still goes on in the Amazon basin (Patiño 1963; Kerr and Clement 1980; Clement 1989a; Clement *et al.*^2009^). Domestication, therefore, is not merely a historical event but a dynamic process that today can be studied in many indigenous communities (Posey 1992) as we demonstrate in this paper.

The anthropogenic factors that affect palm populations include not only forest fragmentation and selective logging (Lowe *et al.*^2005^), but also palm cultivation. The genetic structure of useful palm species has long been subject to human manipulation through domestication pressure (Clement 1999). For example *Oenocarpus bataua* does not possess a homogenous gene pool over long distances, regardless of the lack of a marked physical barriers to gene flow. This shows that the genetic structure of populations is not simply the effect of climatic gradients and wide altitudinal ranges (Montúfar 2007). We suggest that humans may impact the genetic structure of palm populations, but this remains to be investigated in more detail.

According to a review of the management of useful palms in South America (Bernal *et al*. 2011), the enrichment of palm populations by transplanting seedlings and dispersing seeds remains unclear. These practices, which are connected to the traditional ecological knowledge of communities, were reported only in relation to nomadic Amerindians for *Euterpe oleracea* (Goudling and Smith 2007) and *Oenocarpus bataua* (Poltis 1996). The review mentions that *Astrocaryum aculeatum, A. chambira, Euterpe precatoria, Oenocarpus bataua, O. minor* and *Mauritia flexuosa* are generally planted in fallows (e.g., Hammond *et al.*^1995^; de Jong 2001; Miranda *et al.*^2008^; Flores *et al.*^2009^) and that *Attalea colenda, A. speciosa, Bactris gasipaes, Euterpe oleracea, E. precatoria, Mauritia flexuosa, Oenocarpus bataua,* and *Phytelephas aequatorialis* are incorporated in agroforestry systems (e.g., Clement 1989b; Borgtoft and Balslev 1990; Ríos 2001), although it does not investigate palm cultivation practices.

Bernal *et al.* (2011) stress mismanagement in palm harvest practices in South America. They express concern that the practice of cutting down individuals of *Mauritia flexuosa* just to get their fruits is widespread among harvesters, citing examples from Brazil, Colombia, Ecuador, and Peru (Ruiz-Murrieta 1991; Hiraoka 1999; Castaño *et al.*^2007^; Manzi and Coomes 2009). Unfortunately, harvesters in Brazil, where *Mauritia flexuosa* is a very important non-timber forest product (NTFP), do not return seeds to the swamp forest after removal of the fruit pulp (Sampaio *et al.*^2012^). However, studies of the socio-cultural importance of *Mauritia flexuosa* stands for Maijuna communities in Peru reveal that the commercial harvest is only one facet of the relationship between indigenous people and the *Mauritia flexuosa* habitat and resource. *Mauritia flexuosa* stands are important also for game hunting, extraction of other useful plants from this habitat as well as for conservation of traditional ecological knowledge and beliefs (Gilmore *et al.*^2013^). Moreover, local, especially indigenous, inhabitants of the Amazon often gather the fruit by climbing the palms rather than cutting them down (Moreno *et al.*^1991^; de Castro 1993; Zent and Zent 2002; Weinstein and Moegenburg 2004). The Huaorani of Ecuador sometimes plant a *Cecropia* tree near the planted *Bactris gasipaes* to facilitate climbing to reach the palm’s fruits (Borgtoft and Balslev 1990). We did not observe this practice in our study area. However, Asháninka harvest fruits of spiny *Bactris gasipaes* in accordance with their belief in *buen vivir* (well-being), ensuring the survival of the palm.

Studies conducted by Wezel and Ohl (2005) in villages of a neighbouring ethnic group reveal that Matsiguenka home gardens contained cultivated individuals of six palm species: *Bactris gasipaes*, *Mauritia flexuosa*, *Attalea* sp., *Cocos nucifera*, *Oenocarpus bataua* and *Bactris* sp. However, except for *Bactris gasipaes* (the only palm species cultivated also in Matsiguenka fields), palms were not common in Matsiguenka home gardens. They concluded that high availability of many plant resources in the surrounding forests was one of the reasons for relatively low numbers cultivated palms in Matsiguenka home gardens (Wezel and Ohl 2005). It could also be the reason that overall palm cultivation there is rare.

Asháninka have influenced their natural environment to increase availability and accessibility of palm resources. Undoubtedly there are variations in the management of palm resources among different regions within Asháninka territory. Asháninka from the Tambo region have modified palm diversity and distribution in their territory. Their landscape domestication was initiated by opening space for settlements and cultivation of palms, both native and introduced from remote areas and resulted in abundant populations of focal palm species. Social anthropology, through analyses of intermarriage, settlement and customs of seed handing over, can contribute to studies on crop genetic resources in situ (Leclerc and Coppens 2012). This approach is also useful in studies on focal useful palm species because seed exchange is built upon and expands with familiarity and trust among and within communities.

Resource exchange of *Attalea phalerata* and *Phytelephas macrocarpa* among Asháninka fit the general model of plant domestication which, according to Clement *et al.* (2009):

*…* starts as a single founder event: that is, the selection – conscious or not – of a plant or plants for propagation. This founder event may occur in one place at one time or, more rarely, as multiple events in space and time. It becomes a process if the results of the propagation are successful in the eyes of those who initiated it. After the founder event(s), which may already involve movement of seeds from a natural population to a human settlement or other domesticated landscape, seed movement by humans is essential for the domestication process to move forward, because it isolates the next generation from its ancestral population. This isolation reduces gene flow, via pollen or seed dispersal, between the human-propagated and the ancestral populations, and allows the propagated population to diverge from the ancestral population according to human selection criteria.

For example, individuals of *Attalea phalerata* growing in Savareni village possess some characteristics of *Attalea weberbaueri* (Pintaud 2008) distinguished within the *Attalea phalerata* complex. They possess wide leaflets that are preferred for roof thatch, develop only short aerial stems, which facilitate harvesting, and occur only in anthropogenic landscapes.

## Conclusion

Distribution and abundance of the most useful palm species across the Tambo region (*Attalea phalerata—tsiaro, Bactris gasipaes—kiri, Euterpe precatoria—tsirentsi, Iriartea deltoidea—camona, Mauritia flexuosa—toniro, Oenocarpus bataua—shaki, Oenocarpus mapora—chorina*, and *Phytelephas macrocarpa—compiro*) have been and continue to be significantly modified by Asháninka. The most intensively managed palms are those favoured for thatch, and this was reported by respondents as the most important reason for planting *Attalea phalerata*, *Oenocarpus mapora* and *Phytelephas macrocarpa*. Of these, *Attalea phalerata* is the most commonly cultivated palm, and in the Tambo region it is only found in cultivated stands. The palm was introduced to the villages and surrounding fields from already cultivated stands by deliberate seed planting and provide a clear example of ongoing processes of domestication. *Phytelephas macrocarpa* and *Oenocarpus mapora* are both species found in the forest surrounding Asháninka villages, but are planted in home gardens and fields to alleviate limited availability in the wild as well for greater convenience of harvesting.

*Bactris gasipaes,* a domesticated palm that plays an important role in Asháninka culture and everyday life, is planted mainly in fields outside the village. *Mauritia flexuosa’s* swamp habitat is not very widespread in our study area, and its abundance depends mostly on cultivation. *Euterpe precatoria* and *Iriartea deltoidea* grow abundantly in the forest. The harvesting of *Iriartea deltoidea,* which is less abundant near the village, by cutting down the palms slightly influences their distribution.

Their willingness to experiment and openness to new possibilities influence the Asháninka in their selection of known or recommended useful palms for cultivation. For thatch, Asháninka rely on cultivated palms. Most other palm products are also obtained by cultivation (also wood of *Oenocarpus mapora*). However, palm wood is mainly obtained from wild stands of *Iriartea deltoidea.*

Asháninka traditional management heightens palm diversity through the introduction and cultivation of useful palms. All stands of *Attalea phalerata* that we observed in the Tambo region were cultivated, and possessed the characteristics of *Attalea weberbaueri,* described as an endemic species of Peru from the Asháninka management area. Results obtained with ethnobotanical and ecological methods have implication for domestication studies within the *Attalea phalerata* complex (sensu Henderson *et al.*^1995^), using morphometric and genetic methods to compare wild and managed populations. *Attalea weberbaueri* seems to be a semi-domesticated or even domesticated (if time shows that it cannot survive without human intervention) landrace within the *Attalea phalerata* complex.

## Footnotes

1 The former village of Savareni was founded more than 30 years ago on the western side of the Tambo River by three women from the Kushiviani community of Rio Negro. In 1993, during the war with the Shining Path (1980–2000), families that lived near the Savareni stream were forced away. They left all their stock, which were slaughtered by the Shining Path, and settled on Tambo Island. Later fields were established on the island, which remain in use today. After an elderly woman was nearly shot near the Savareni stream, she decided to move the group to the eastern side of Río Tambo, the current location of Savareni. This location was previously occupied by an Asháninka family, which left the place after the death of a family member.

2 The *Phytelephas macrocarpa* palm seeds were planted in threes in every seedbed over a 50 × 100 m field. The family planned to build an additional house, which they completed ten years later even though the palm leaves were considered ready for harvest five years after planting.
